# Investigating the beneficial effect of aliskiren in attenuating neuropathic pain in diabetic Sprague‐Dawley rats

**DOI:** 10.1002/edm2.209

**Published:** 2020-11-25

**Authors:** Shouq Alkhudhayri, Rania Sajini, Bashayer Alharbi, Jumana Qabbani, Yosra Al‐Hindi, Arwa Fairaq, Amal Yousef

**Affiliations:** ^1^ Faculty of pharmacy University of Umm Al‐Qura Makkah Saudi Arabia; ^2^ Faculty of Pharmacy University of Umm Al‐Qura Makkah Saudi Arabia; ^3^ Faculty of Medicine Cairo University Giza Egypt

**Keywords:** aldose reductase activity, aliskiren, antioxidant activity, diabetes, gliclazide, neuropathy, ramipril, renin‐angiotensin system, Sprague‐Dawley rats

## Abstract

**Objectives:**

Worldwide, diabetic neuropathy (DN) is a major complication of diabetes mellitus. The direct renin inhibitor aliskiren is recognized as a treatment for cardiovascular disease in diabetic patients, but little is known about its potential benefits in cases of diabetic neuropathy. Accordingly, we investigated the effects of aliskiren (ALIS) and gliclazide (GLZ) and their combination therapy on peripheral neuropathy in streptozotocin‐induced diabetic rats.

**Methods:**

In total, 112 adult Sprague‐Dawley rats were used for this study. Diabetes was induced using streptozotocin (STZ), whereas the control group was treated with an equal volume of citrate buffer. The diabetic rats were divided randomly into six groups according to the proposed treatment regime: diabetic control (DC), gliclazide (GLZ), aliskiren (ALIS), ramipril (RAM), (GLZ + ALIS) and (GLZ + RAM). Behavioural responses to thermal (hot‐plate) and mechanical (tail‐pinch) pain were evaluated. After eight weeks of daily treatments, the animals were fasted and sacrificed. The blood samples were collected, with the serum separated and subjected to various biochemical and enzyme analyses so as to assess the effect of the treatments on diabetic peripheral neuropathy.

**Results:**

After 8 weeks, aliskiren alone and in combination with gliclazide therapy had a significant effect (*P* < .001) in reducing blood glucose levels and showed increased hot‐plate and tail‐flick latencies compared with the diabetic control group. The threshold of mechanical hyperalgesia was also significantly elevated (*P* < .001).

**Conclusions/Interpretations:**

These data suggest that either aliskerin alone or in combination with gliclazide can protect against the development and progression of diabetic neuropathy.

## Graphical abstract

The combination of aliskiren with gliclazide can protect effect against the development and progression of diabetic neuropathy. The combination of aliskiren with gliclazide can decrease blood glucose levels in persons who have diabetes.

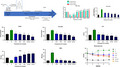

**What is already known about this subject?**

Diabetic neuropathy (DN) is a major complication of diabetes mellitus. The direct renin inhibitor aliskiren is recognized as a treatment for cardiovascular disease in diabetic patients.

**What is the key question?**

Does aliskiren have a protective effect in diabetic neuropathic pain?

**What are the new findings?**

The use if of aliskiren as monotherapy or in combination with gliclazide can protect effect against the development and progression of diabetic neuropathy.

The use if of aliskiren as monotherapy or in combination with gliclazide can decrease blood glucose levels in persons who have diabetes.

**How might this impact on clinical practice in the foreseeable future?**

Aliskiren can protect against the development and progression of diabetic neuropathy.

## 1 BACKGROUND

According to the World Health Organization (WHO), Saudi Arabia has the second‐highest diabetes prevalence in the Middle East and the seventh‐highest globally.^1^ Moreover, around 7 million people were estimated to be diabetics and nearly 3 million are prediabetics.^2^ Diabetes is associated with high levels of mortality and morbidity as well as complications of the cardiovascular system and overall low quality of life. In Saudi Arabia, diabetes is becoming increasingly commonly and is a major cause of medical complications and even death.^1^

Neuropathic pain is defined by the International Association for the Study of Pain (IASP) as ‘pain caused by a lesion or disease of the somatosensory nervous system’. It can be caused by the effects of certain diseases, such as diabetic neuropathy (DN), human immunodeficiency virus/AIDS and injury to the peripheral or central nervous system.^2^

Diabetic neuropathy is a progressive condition that can cause damage to the nerves as a result of high glucose and increased level of lipids in the blood. The symptoms vary with the type of nerve damage and can affect peripheral nerves (legs) or internal organs (heart). Multiple studies have elucidated that the prevalence of DN is increasing globally. A study was done in Netherland showed that this DN rate is increasing with the increase in age of diabetic patients.^3^ Moreover, multiple studies have shown that DN rate is related more with diabetes type 2 rather than type1.^4^, ^5^, ^6^ Furthermore, the prevalence of DN in Middle‐Eastern diabetic populations is relatively high. For example, in Bahrain, a neighbouring country of Saudi Arabia, the prevalence rate is almost 36.6% that been reported in a large diabetic population,^7^ comparable to that in US diabetic populations which as between 12% and 50%.^8^ Moreover, in Saudi Arabia, the prevalence is between 19.9% and 35%.^9^

The renin‐angiotensin system (RAS) plays an important role in hypertension, fluid volume and electrolyte balance.^10^ However, inhibition of the RAS system has also been reported to attenuate pain for those who have certain conditions, such as migraine, nociceptive pain and pancreatic pain.^11^ Aliskiren is a drug that inhibits the activity of renin directly, blocking the renin angiotensin aldosterone system (RAAS) and subsequently reducing the production of angiotensin I and II and aldosterone. We found little information about review studies that have demonstrated the usefulness of aliskiren in treating neuropathy pain. The researchers believed that in the future, RAAS modulators can play a significant role in managing neuropathic pain and other neurodegenerative disorders such as Parkinson disease and amyotrophic lateral sclerosis. However, more extensive clinical research is needed into the use of RAAS modulators in cases Our study is unique in examining aliskiren's ability to attenuate neuropathic pain in diabetic‐induced rats. The American Diabetes Association recommends medicines for the relaxation of signs and symptoms associated with DN that have been proven to enhance patient's life.^7^ Currently, duloxetine and pregabalin are permitted through the United States Food and Drug Administration (FDA) for the remedy of DN.^7^, ^8^ Tricyclic antidepressants were proven to lessen neuropathic ache, however, aren't presently permitted through the FDA for this indication in large part because of their threat of adverse effects.^12^ Moreover, pain control with tramadol or oxycodone has additionally been proven to decrease ache ratings and enhance bodily characteristic in a few patients.^9^ However, opioids have addictive problem and ought to now no longer be used as first‐ or second‐line remedy for this neuropathic ache.^11^ Therefore, there is an urgent need to investigate other therapeutic options to ensure both efficacy and safety, especially when we require a combined treatment with an oral antidiabetic drug to prevent the development of peripheral diabetic neuropathy. We chose gliclazide based on evidence from previous work that concluded that the combined administration of curcumin with gliclazide showed marked increase in the nociceptive threshold of pain.^13^ We also added Ramipril based on an evidence from a study that concluded that ramipril significantly attenuated chronic constriction injury‐induced rise in peripheral and central pain sensitivity.^2^ Accordingly, new treatments are needed for successful neuropathic pain management of neurodegenerative disorders.^14^

## 2 METHODS

### 2.1 Animals

All animal studies and procedures were approved by the Animal Care and Use Committee of Umm Al‐Qura University and were conducted in accordance with the guidelines for the care and use of laboratory animals.^15^ Six‐week‐old adult male Sprague‐Dawley rats weighing 280‐300 g were purchased from the animal breeding unit Harlan (Charles River Laboratories, Wilmington, Massachusetts, USA) and Jackson Laboratories (Bar Harbour, ME, USA) and were housed in plastic cages (three to four rats per cage) under conventional laboratory conditions throughout the period of investigation (8 weeks). Rats were acclimatized for 2 weeks and maintained in a well‐ventilated animal house, on a 12:12h light:dark cycle. Animals were fed a standard pellet diet and given free access to water. A small‐scale pilot experiment was conducted at the beginning of the study to assess the antinociceptive effects of aliskiren, ramipril and gliclazide per se, using the hot‐plate, tail‐flick and tail‐pinch pain tests in control animals,we found no significant effect between the control and the drug‐treated animals (data not shown).

### 2.2 Induction and assessment of diabetes

**FIGURE 1:**
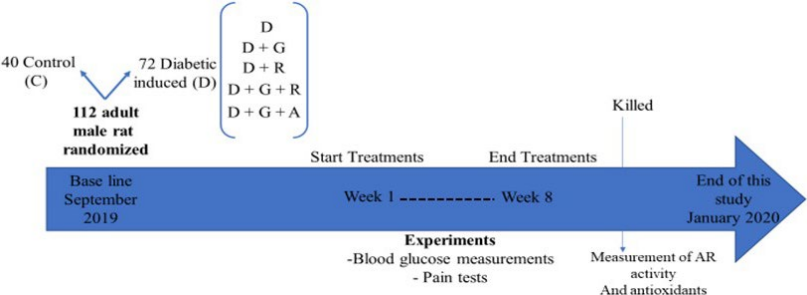
Schematic presentation of the study methodology

Diabetes was induced in overnight‐fasted rats by a single dose of streptozotocin (45 mg/kg, intraperitoneally), dissolved in cold citrate buffer (0.1 M, pH 4.5).^8^ This dose was selected to cause incomplete destruction of pancreatic beta cells, based on reports that streptozotocin can produce mild to severe types of diabetes, depending on the dose, when administered to adult rats by either a single intravenous or an intraperitoneal injection.^16^ After the injection, the rats had free access to food and water and were given a 5% glucose solution to drink overnight so as to counter hypoglycaemic shock. Age‐matched and weight‐matched control rats received citrate buffer at the identical amount and pH. Diabetes was confirmed by measuring the fasting tail blood glucose concentration using a glucose meter device (Accu‐chek Go, Roche Diagnostics GmbH, Mannheim, Germany) 96 h after injection with streptozotocin. Ninety‐nine per cent of the animals had blood glucose above 235 mg/dL and were considered to be diabetics,rats with blood glucose below 235 mg/dL were excluded from the present study (Figure 1).

### 2.3 Experimental design and sample size

We calculated the sample size according to when end‐point is in qualitative scale such as alive/death, diseased/nondiseased, male/female and pain/no pain; then, this formula can be used for sample size calculation for comparison between two groups.

Sample size = 2 (Za/2 + Zβ) 2 × P (1 − P)/(p1 − p2) 2Za/2 = Z0.05/2 = Z 0.025 = 1.96 (From Z table) at type 1 error of 5%Zβ = Z0.20 = 0.842 (From Z table) at 80% powerp1 − p2 = Difference in proportion of events in two groups P = Pooled prevalence = (prevalence in case group [p1] + prevalence in the control group [p2])/2. The sample size was around 16 animal per group and 2 extra animals for spare in case anything occurs.

Animals were randomly classified into seven groups (*n* = 16) and received their respective drug treatments daily for 8 consecutive weeks according to the following design:

Group 1: control animals that received distilled water orally and citrate buffer intraperitoneally (C)
Diabetic animals were subclassified into the following six groups:
Group 2: untreated diabetic animals that received streptozotocin (45 mg/kg, intraperitoneally) and served as a diabetic control (D) group
Group 3: diabetic rats that received gliclazide (25 mg/kg, orally; [10]), (D + G) group
Group 4: diabetic rats that received ramipril (8 mg/kg, orally;[12]), (D + R) group
Group 5: diabetic rats that received aliskirine (45 mg/kg, orally;[11]), (D + A) group
Group 6: diabetic rats that received both gliclazide (25 mg/kg, orally) and ramipril (8 mg/kg, orally) (D + G+R) group
Group 7: diabetic rats that received both gliclazide (25 mg/kg, orally) and aliskirine (45 mg/kg, orally)

## 3 ASSESSMENT

### 3.1 Blood glucose measurements

Blood glucose was measured using a One Touch II glucose meter (Lifescan, Inc, Johnson & Johnson), and this was done every two weeks until the end of the study.

### 3.2 Pain tests

During the acclimatization period, the normal responses of the animals to pain stimuli (baseline latencies) were determined twice every week.

Thermal hyperalgesia was assessed by measuring nociceptive thermal thresholds using a hot‐plate analgesia meter (Harvard Apparatus) and an automated tail‐flick analgesia meter unit (Muromachi, Kikai Co. Ltd).

In the hot‐plate test, the apparatus was adjusted to 52.5°C and the baseline reaction time was between 10 and 12 seconds (s). Rats were examined for latency (s) to lick their hind paw or jump from a noxious thermal stimulus. The cut‐off time was set at 15 s to avoid tissue damage. Values were recorded twice for each animal, with a 10‐min interval between tests.^17^

For the tail‐flick test, the heat latency response was measured as the reaction time in seconds to remove the tail from the source of noxious radiant heat. The intensity of the light beam was adjusted so that the baseline reaction time was 15 s, and the cut‐off time was set at 20 s to avoid tissue damage. The light beam was focused on the same spot, about 3 cm from the tip of the tail. The average threshold of the control response was determined by a total of two consecutive measurements separated by a 10‐min interval.^18^

Mechanical hyperalgesia was assessed using the tail‐pinch method. Rats were placed in a clean cage, and an alligator clip exerting a force equal to 625 g of weight was placed approximately 5 cm from the tip of the tail.^19^ The latency for vocalization, biting or flicking the clip was recorded. Baseline latencies were between 10 and 15 s with a 25 s cut‐off. The mean threshold of the control response was determined by a total of three consecutive measurements separated by 10‐min intervals.

### 3.3 Sample collection of the sciatic nerve

After the animals were anesthetized and killed, their sciatic nerves were dissected rapidly at the endpoint of the experiment. The right sciatic nerves were weighed and prepared for enzyme assays. A 5‐mm section from the proximal part of the left sciatic nerves was removed and fixed with 4% (v/v) formaldehyde for histopathological examination. The rest of the left sciatic nerve tissue was flash‐frozen in liquid nitrogen and stored at −70°C until used for the reverse‐transcription (RT) polymerase chain reaction (PCR). Each right sciatic nerve was homogenized on ice in 1.5 mL of ice‐cold 0.9% (w/v) NaCl solution and centrifuged at 1050 × g for 10 min at 4°C. The total protein content in the supernatant was quantified using the Bradford assay to equalize protein concentration with 0.9% NaCl solution and was then used for measurements and enzyme activity assays.

### 3.4 Measurement of aldose reductase (AR) activity in the sciatic nerve

AR activity was measured using the method reported by Ohtaka.^20^ Briefly, 20 μL of sciatic nerve supernatant was incubated with 135 mmol/L Na,K‐phosphate buffer (pH 7.0), 0.1 mol/L Li2SO4, 0.1 mmol/L DL‐glyceraldehyde and 0.03 mmol/L NADPH in a volume of 1 mL at 37°C for 10 min. The reaction was terminated by the addition of 0.3 mL of 0.5 mol/L HCl,1 mL of 6 mol/L NaOH containing 10 mmol/L imidazole was then added. Fluorescent NADP was measured by fluorescence spectrophotometry (MPF‐4, Hitachi, Japan) at an excitation wavelength of 366 nm and an emission wavelength of 455 nm. AR activity was expressed as the depletion of nM NADPH/min/mg of protein.

### 3.5 Assay of antioxidative enzyme activities, peroxidation products and antioxidant components in sciatic nerve tissues

The activities of antioxidative enzymes, including superoxide dismutase (SOD), catalase (CAT) and glutathione peroxidase (GSH‐Px), as well as the contents of the tissue lipid peroxidation product malondialdehyde (MDA) and antioxidant component GSH, were measured following the manufacturer's instructions for the assay kits (Nanjing Jiancheng Biotech, China).

### 3.6 Statistical analysis

All results are expressed as group means ± SEM. Results were analysed by one‐way analysis of variance, followed by Tukey's post hoc test to assess significance, using a criterion of *P* < .05. The statistical analysis was carried out using GraphPad Prism version 5 (GraphPad Software Inc).

## 4 RESULTS

### 4.1 Pain tests

**FIGURE 2:**
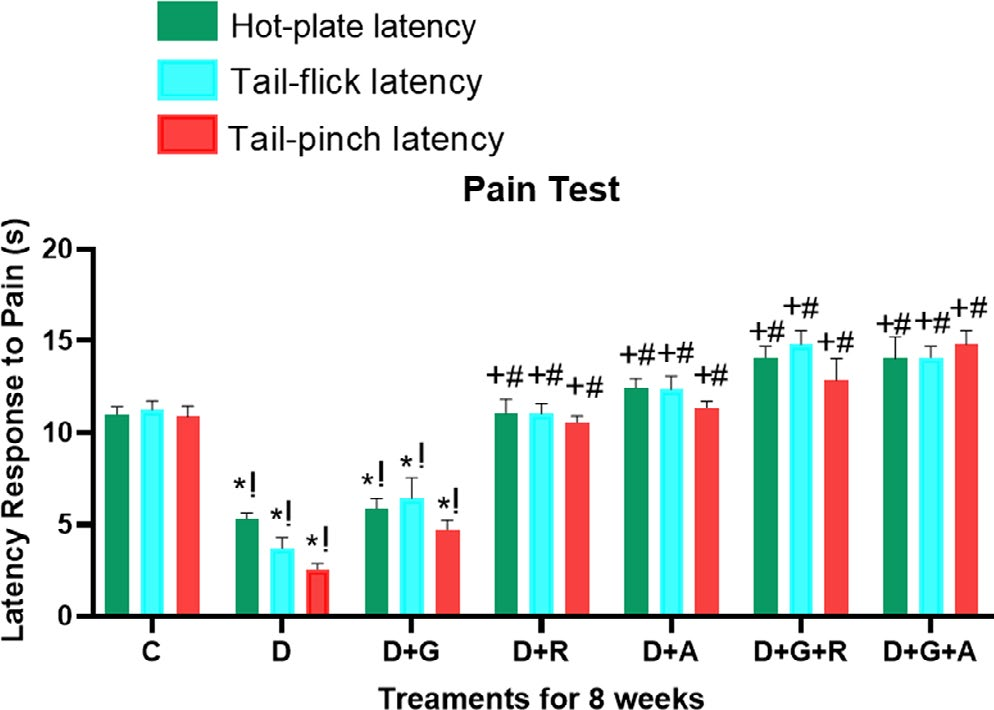
Effect of aliskiren and ramipril and their combination with gliclazide on thermal (hot‐plate/tail‐flick) and mechanical (tail‐pinch) hyperalgesia in streptozotocin‐diabetic rats. ^*^ *P* < .05 relative to control (C); ^+^ *P* < .05 relative to diabetics (D); ^#^ *P* < .05 relative to gliclazide (D + G); ^!^ *P* < .05 relative to gliclazide–aliskiren (D + G+A)

Streptozotocin‐induced diabetic rats showed increased levels of response to pain when thermal and mechanical stimuli were applied in comparison with the control group, as measured by the hot‐plate, tail‐flick and tail‐pinch tests (Figure 2). This was rational with the early neuropathy criteria that can be developed in the diabetics.^21^

In the hyperalgesic response on the hot plate, which is considered to result from a combination of central and peripheral mechanisms, the post hoc analysis showed that streptozotocin animals that was treated showed a 55% decrease in the threshold of pain in comparison with the control animals. Oral administration of monotherapies of gliclazide (25 mg/kg, orally) did not show a significant improvement; however, ramipril (8 mg/kg, orally) and aliskiren (45 mg/kg, orally) for 8 consecutive weeks did show a better rate of response to pain. However, the combined treatment with gliclazide with ramipril and gliclazide with aliskiren for the same treatment period showed a significant elevation to the pain threshold (*P* < .05) in comparison with the diabetics controlled group. But it was not that different from using aliskiren and ramipril alone.

### 4.2 Biochemical measures

**FIGURE 3:**
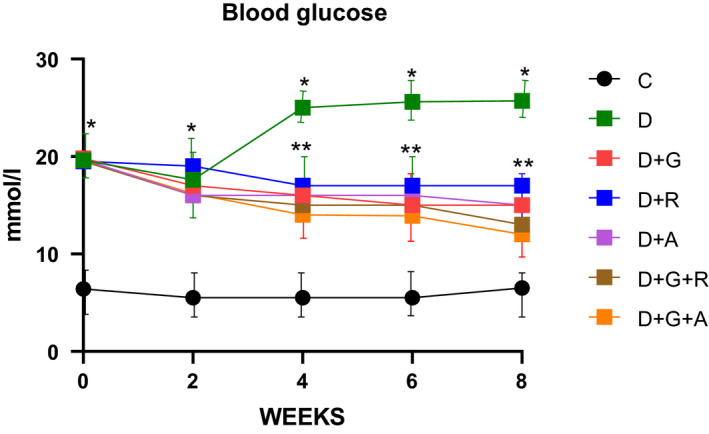
Effect of aliskiren and ramipril and their combination with gliclazide on blood glucose levels in streptozotocin‐diabetic rats. ^*^ *P* < .05 relative to control (C); ^**^ *P* < .05 relative to diabetics (D)

**FIGURE 4:**
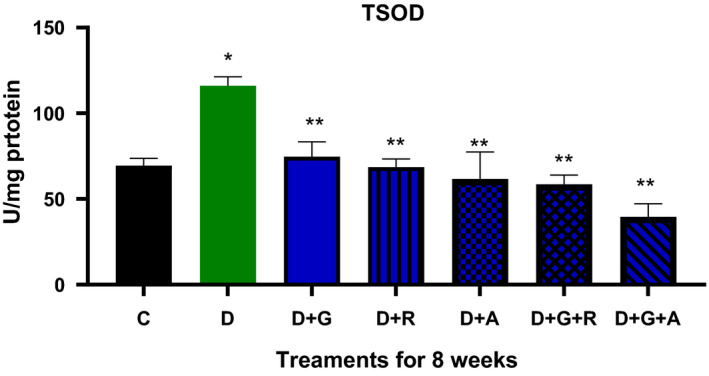
Effect of aliskiren and ramipril and their combination with gliclazide on TSOD activity of sciatic nerve in streptozotocin‐diabetic rats. ^*^ *P* < .05 relative to control (C); ^**^ *P* < .05 relative to diabetics (D)

**FIGURE 5:**
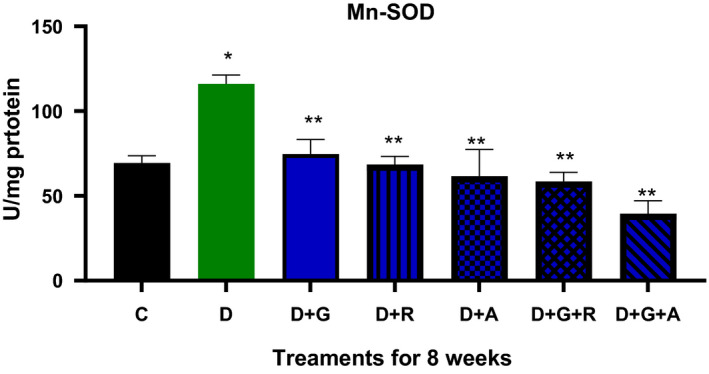
Effect of aliskiren and ramipril and their combination with gliclazide on MnSOD activity of sciatic nerve in streptozotocin‐diabetic rats. ^*^ *P* < .05 relative to control (C); ^**^ *P* < .05 relative to diabetics (D)

**FIGURE 6:**
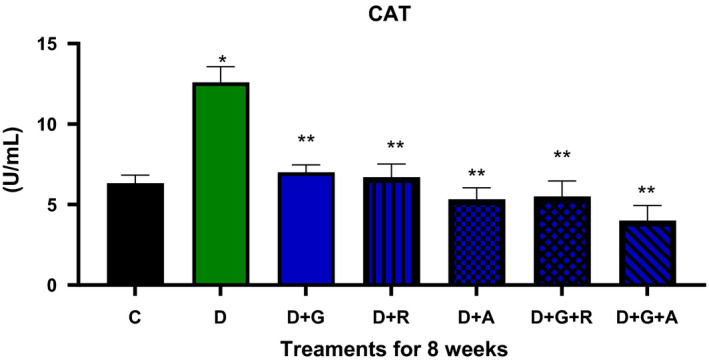
Effect of aliskiren and ramipril and their combination with gliclazide on CAT activity of sciatic nerve in streptozotocin‐diabetic rats. ^*^ *P* < .05 relative to control (C); ^**^ *P* < .05 relative to diabetics (D)

**FIGURE 7:**
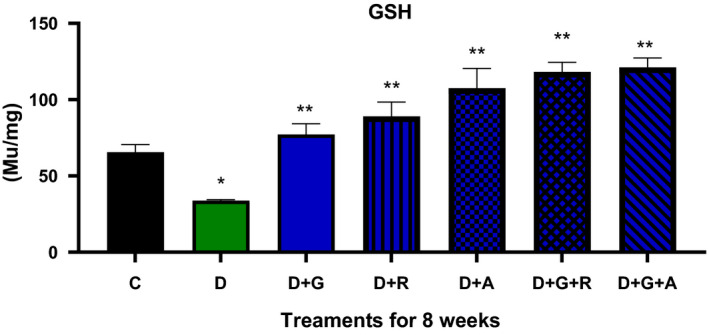
Effect of aliskiren and ramipril and their combination with gliclazide on GSH activity of sciatic nerve in streptozotocin‐diabetic rats. ^*^ *P* < .05 relative to control (C); ^**^ *P* < .05 relative to diabetics (D)

**FIGURE 8:**
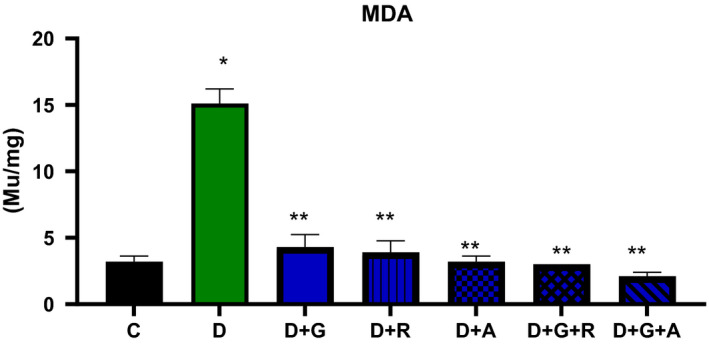
Effect of aliskiren and ramipril and their combination with gliclazide on MDA activity of sciatic nerve in streptozotocin‐diabetic rats. ^*^ *P* < .05 relative to control (C); ^**^ *P* < .05 relative to diabetics (D)

Diabetic animals showed a marked elevation in the serum of glucose levels (Figure 3); this was accompanied by a significant elevation in TSOD, Mn‐SOD, CAT and MDA activities and a significant decrease in GSH levels (Figures 4, 5, 6, 7, 8).

**FIGURE 9:**
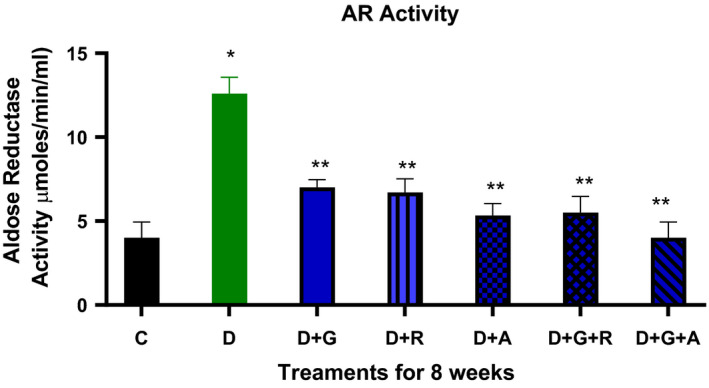
Effect of aliskiren and ramipril and their combination with gliclazide on aldose reductase activity of sciatic nerve in streptozotocin‐diabetic rats. ^*^ *P* < .05 relative to control (C); ^**^ *P* < .05 relative to diabetics (D)

The treatment of gliclazide, ramipril and aliskiren as monotherapy showed a significant effect on the reduction glucose levels. However, the effect of gliclazide and aliskiren (D + G + A) combination showed a marked significant decrease compared to the control diabetic animals. Similarly, this treatment combination also showed a significantly decrease in the AR activity (*P* < .05) (Figure 9), TSOD, Mn‐SOD, CAT and MDA activities, while there was a significantly increase in GSH activity (Figures 4, 5, 6, 7, 8).

## 5 DISCUSSION

The results clearly demonstrated a role for aliskiren in attenuating diabetes‐related neuropathic pain. To our knowledge, no published data have reported an effect of aliskiren on neuropathic pain; our study is the first to have investigated aliskiren's ability to attenuate diabetic neuropathic pain in diabetic‐induced rats. We compared the effect of aliskiren in normal rats and diabetic‐induced rats.

When we evaluated the effect of aliskiren on pain response using different combinations, we noticed an improvement when aliskiren was administered either alone or in combination with gliclazide in diabetic‐induced rats. Subsequently, we evaluated the effect of aliskiren on antioxidant activities, which plays an important role in neuropathic pain. Multiple studies demonstrated that oxidative stress is essential process in many neurological disorders.^10^ It was found that antioxidant activities are involved in the nociceptive process.^11^ Moreover, the involvement of reactive oxygen species (ROS) on this manner has been reported in previous researches.^13^, ^22^ ROS entails several nonenzymatic molecules and enzymes inclusive of superoxide dismutase (SOD) and catalase. Therefore, investigating the probable association between neuropathic pain and alterations in the activity and expression of antioxidant enzymes such as SOD and catalase seems vital and reasonable.

As a result, we investigated different combinations and tested different types of antioxidants such as TSOD, Mn‐SOD, Cat, GSH and MDA. We found more marked improvements with the combination of aliskiren than with other combinations on TSOD, Mn‐SOD and Cat. Moreover, we saw a significant increase in the GSH enzyme, which features in the natural defence pathway against diabetic neuropathy.^21^ We also evaluated different combinations’ effect on aldose reductase activity and found significant improvements when aliskiren and gliclazide were used as a combination. During the study, we measured blood glucose levels every two week, which was decreased when aliskiren was used alone or in combination with gliclazide.

Several studies have investigated aliskiren's protective effects against various types of pain, but no other studies have examined its ability to attenuate neuropathic pain in the laboratory on diabetic‐induced rats. One review described current perspectives on RAAS in the management of neuropathic pain in humans and animals. The researchers saw a significant future role for RAAS modulators in managing neuropathic pain and other neurodegenerative disorders, such as amyotrophic lateral sclerosis and Parkinson disease. However, use of RAAS modulators in neurodegenerative disorders required more extensive clinical research.^10^ The dual role of the RAAS in different states of pain was described in another review, in which researchers found that RAAS had a role in modulating different physiological/pathological functions, including pain. Moreover, beneficial effects have been reported in cases of migraine and neuropathic and nociceptive pain for drugs that block RAAS activation versus renin inhibitors.^11^ Another study demonstrated that aliskiren is dose‐dependent and has a potential effect as an antinociceptive and antiallodynic agent in chemically induced pain, orofacial pain and centrally mediated pain models. In postoperative and neuropathic pain models, aliskiren was used intraperitoneally in Sprague‐Dawley rats and antiallodynic activity was assessed (30‐100 mg), revealing that chronic constriction causes antiallodynic activity in postoperative pain and neuropathic pain.^4^ Another study found that in cases of chronic constriction injury (CCI), administration of aliskiren (25 and 50 mg/kg intraperitoneally in Institute of Cancer Research [ICR] mice) for 14 days significantly attenuated neuropathic pain. The researchers found that in cases of nerve injury, as well as chronic pain and, especially, neuropathic pain, evidence suggested that manipulating RAAS could be beneficial. For this reason, we chose aliskiren.^23^, ^24^, ^25^ Although metformin is the first choice for treatment of diabetes, we chose gliclazide for our study because metformin can cause vitamin B12 deficiency, whereas gliclazide has a neuroprotective effect.^9^ One study that investigated the effect of a combination of aliskiren with metformin did report an improvement in neuropathic pain,^17^ but this finding needs further investigation. A study used the combination of gliclazide as and curcumin showed a marked increase in the nociceptive threshold to thermal (hot plate and tail flick) and mechanical (tail pinch) pain with respect to the diabetic group.^17^ We used Ramipril on the basis of a study showed that the administration of ramipril (2 and 4 mg/kg, *p.o*.) significantly attenuated chronic constriction injury‐induced rise in peripheral and central pain sensitivity (thermal and mechanical) along with impairment of motor in‐coordination activity. Further, it also produced ameliorative effects on chronic constriction injury‐induced rise in thiobarbituric acid reactive substances and decrease in glutathione levels when compared with a normal control group.^18^

Our study had certain limitations: We had too few sciatic nerve samples to allow us to conduct light microscopic observation with immunohistochemical analysis. In future, we can continue our investigations by seeking to understand the exact pathways through which aliskiren decreases blood glucose levels and can also investigate the use of metformin in combination with aliskiren to attenuate neuropathic pain in diabetic patients. In summary, our present findings highlight the neuroprotective effect achieved through either using aliskiren as a monotherapy or in combination with gliclazide in cases of diabetes.

## CONFLICT OF INETREST

Authors state no any conflict of interest.

## AUTHOR CONTRIBUTION

AY, YA and AF conceived and designed the study. YA, SA, RS, BA and JQ acquired and analysed the data. SA, RS and BA contributed to the analysis and interpretation of the data. SA drafted the manuscript. RS, BA, JQ, AY and AF made critical revisions to the manuscript to add important intellectual content. All authors approved the final version of the manuscript to be published. YA is the guarantor of this work.

## AUTHORS’ RELATIONSHIPS AND ACTIVITIES

The authors declare that no relationships or activities might bias, or be perceived to bias, their work.

## Data Availability

The data sets generated and/or analysed during the current study are available from the corresponding author on reasonable request.
